# Control of Tyrosine Kinase Signalling by Small Adaptors in Colorectal Cancer

**DOI:** 10.3390/cancers11050669

**Published:** 2019-05-14

**Authors:** Rudy Mevizou, Audrey Sirvent, Serge Roche

**Affiliations:** CRBM, CNRS, Univ. Montpellier, “Equipe labellisée Ligue Contre le Cancer”, F-34000 Montpellier, France; rudy.mevizou@crbm.cnrs.fr (R.M.); audrey.sirvent@crbm.cnrs.fr (A.S.)

**Keywords:** cell signalling, tyrosine kinase, small adaptor proteins, colorectal cancer, targeted therapy

## Abstract

Tyrosine kinases (TKs) phosphorylate proteins on tyrosine residues as an intracellular signalling mechanism to coordinate intestinal epithelial cell communication and fate decision. Deregulation of their activity is ultimately connected with carcinogenesis. In colorectal cancer (CRC), it is still unclear how aberrant TK activities contribute to tumour formation because TK-encoding genes are not frequently mutated in this cancer. In vertebrates, several TKs are under the control of small adaptor proteins with potential important physiopathological roles. For instance, they can exert tumour suppressor functions in human cancer by targeting several components of the oncogenic TK signalling cascades. Here, we review how the Src-like adaptor protein (SLAP) and the suppressor of cytokine signalling (SOCS) adaptor proteins regulate the SRC and the Janus kinase (JAK) oncogenic pathways, respectively, and how their loss of function in the intestinal epithelium may influence tumour formation. We also discuss the potential therapeutic value of these adaptors in CRC.

## 1. Colorectal Cancer

Colorectal cancer (CRC) is one of the leading causes of malignancy-related death worldwide. Most of these cancers are sporadic and under the control of genetic, epigenetic and environmental factors. While localised tumours can be cured by surgery combined with adjuvant chemotherapy, patients with metastatic tumours have a poor prognosis with a 5-year survival rate of 10% [1,2]. Therefore, effective antimetastatic therapies are still needed for this cancer. Therapeutic failure is associated with metastatic spread. In this process, cancer cells escape the primary tumour, disseminate in the circulation and establish secondary lesions in distant organs, mostly in liver and lungs. Metastatic cancer cell behaviour is characterised by aberrant activity of different signalling pathways that promote their invasive properties and tumour-initiating capacities (also named cancer stems cells, or CSCs), which are under the control of the tumour microenvironment [3]. Among the various oncogenic pathways, the tyrosine kinase (TK) signalling cascades have emerged as important determinants of metastasis development. Consistent with this idea, therapies to target the receptor TKs (RTKs) epidermal growth factor receptor (EGFR) or vascular endothelial growth factor receptor (VEGFR) have been developed for metastatic CRC and are currently used in the clinic [4]. However, they prolong patient survival only by several months and many patients with CRC, including those with cancer displaying dysregulated RAS signalling, do not respond to anti-EGFR therapies [5]. Therefore, a better understanding on how TK signalling promotes metastasis is needed for developing effective therapies and improving the stratification of patients with metastatic CRC.

In an effort to better stratify CRC, extensive genomic analyses have been performed using combined cohorts of CRC specimens. These studies led to the characterisation of four consensus molecular subtypes (CMS1–4) with distinctive features [6]. CMS1 tumours (14%) are hypermutated, are microsatellite instable (MSI+), display strong immune activation and have good prognosis. CMS2 tumours (37%) show epithelial features, high WNT and MYC signalling activation and high proliferative rates. CMS3 tumours (13%) display epithelial feature and metabolic dysregulation and include most KRAS-mutated tumours. Finally, CMS4 tumours (23%) display mesenchymal features with prominent transforming growth factor-β (TGF-β) activation, integrin signalling, stromal invasion and angiogenesis and poor prognosis. Mitogen-activated protein kinase (MAPK) signalling is mainly induced in CMS1 and 3 and RTK signalling in CMS2. It should be noticed that this classification represents more than 80% of the analysed CRC tumours and the others could represent mixed phenotypes.

## 2. TK Signalling in CRC

TKs use protein phosphorylation on tyrosine residues as an intracellular signalling mechanism to coordinate fate decision and cell communication during different cellular processes, such as growth, adhesion, migration, survival and differentiation [7,8]. Mechanistically, intracellular signalling is initiated by TK activation followed by substrate phosphorylation on a specific tyrosine residue that enables the interaction with an SRC Homology 2 (SH2)-domain-containing protein for signal propagation. This signalling mechanism is under the control of tyrosine phosphatases that dephosphorylate the substrates [7,8]. The human proteome contains 90 TKs, including receptors for growth factors and factors involved in cell adhesion and motility, cell survival and metabolism as well as cytoplasmic TKs (CTKs) that transmit signals from RTKs and receptors devoid of TK activity. It also contains about 120 SH2-containing proteins, mainly enzymes and small adaptors, and about 50 tyrosine phosphatases [9]. Due to its central role in cell communication in multicellular organisms, this phosphorylation-dependent mechanism has been conserved during evolution and is highly regulated in normal conditions, as shown by the physiological level of protein tyrosine phosphorylation in human cells (2–3% of the total protein phosphorylation). Deregulation of this kinase pathway often leads to pathological states, such as cancer [10]. However, it is still unclear how its deregulation can induce tumour formation. In principle, aberrant protein tyrosine phosphorylation can result from TK deregulation, aberrant substrate expression/activity and inhibition of protein tyrosine phosphatases. Currently, most of our knowledge of human pathology concerns TK deregulation. Specifically, more than 50% of these TKs are deregulated in human cancer through amplification, fusion or somatic mutation of their corresponding genes [10]. It is thought that these genetic alterations are the molecular causes of TK-dependent tumour formation. The proof of this concept has been provided by the remarkable antitumour activity of the small BCR (breakpoint cluster region)-ABL (Abelson) TK inhibitor imatinib in chronic myeloid leukaemia (CML) [11]. Consequently, these genetic alterations, such as the appearance of a BCR-ABL fusion gene, serve as biomarkers to predict antitumour activity of this inhibitor. However, other TK inhibitors have shown variable effects in cancer, suggesting that TK genetic alteration alone may not be always sufficient to induce oncogenesis and to predict tumour response to these targeted therapies [12].

**Figure 1:**
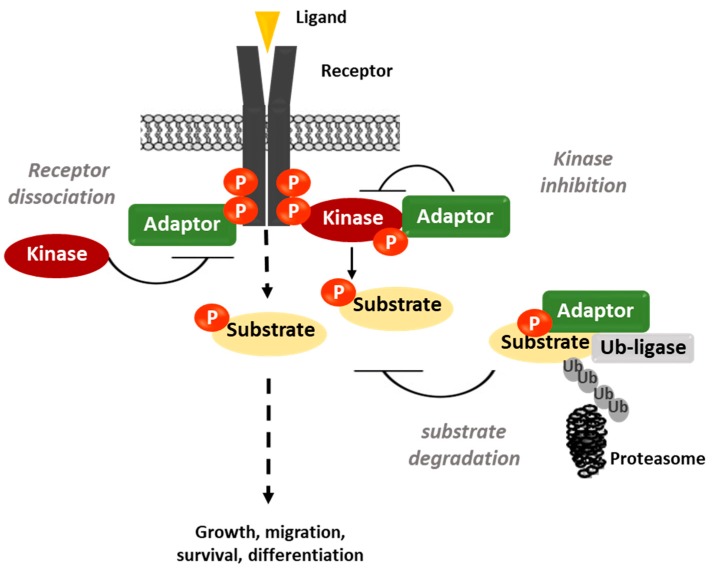
A model showing the negative control of tyrosine kinase (TK) signalling by small adaptors. Adaptor proteins can inhibit TK signalling by competing with effectors for receptor binding, directly inhibiting cytoplasmic TK (CTK) activity or promoting substrate/CTK/receptor degradation via association with specific ubiquitination factors. Ub, ubiquitin; P, phosphorylation.

In CRC, RTKs of the EGFR, VEGFR, ephrin (EPHs), discoidin domain receptor (DDR) and MET hepatocyte growth factor receptor families, and CTKs of the SRC, focal adhesion kinase (FAK) and Janus kinase (JAK) families have been linked to cancer progression; however, the genes encoding these TKs are not frequently mutated or amplified in these tumours [13,14,15]. Unlike CML, the level of TK expression or activity is not a good predictor of therapeutic response in CRC. For instance, EGFR is expressed in most CRCs and its expression level is a marker of poor prognosis [16], but the clinical activity of anti-EGFR antibodies does not correlate with its tumour protein level [17]. Therefore, important, yet unknown genetic-independent mechanisms may be involved in TK activation in CRC. Interestingly, during evolution, the activity of several TKs has become tightly controlled by small adaptor proteins that act as a fine-tuning mechanism [18]. These cytosolic proteins lack intrinsic catalytic activity and signal by linking two functional members of a catalytic pathway. While most small adaptors display positive regulatory functions [9,18], a small group exerts negative regulatory functions by targeting several components of the TK signalling cascade [18]. Probably, the most prominent and ancient example is the suppressor of cytokine signalling (SOCS) family that targets the immune-responsive JAK/signal transducer and activator of transcription (STAT) pathways [18]. Mechanistically, they act via sophisticated mechanisms that implicate competition with effectors/substrates for TK binding, direct inhibition of TK activity or substrate/TK degradation via its association with specific ubiquitination factors (Figure 1). Genetic analyses in mice highlighted important roles for this adaptor-based regulatory mechanism during haematopoiesis and immunity (for review, see [18,19]). However, their role in nonhaematopoietic tissues is largely unexplored. Interestingly, this mechanism can play important roles upon aberrant induction of TK signalling, as illustrated by their prominent tumour-suppressor activity in human cancers [18,20]. Here, we describe the important role the Src-like adaptor protein (SLAP) and SOCS families of adaptors in the control of two major oncogenic pathways initiated by SRC and JAK activities in CRC. We also discuss the potential prognostic and therapeutic value of these adaptors in CRC.

## 3. The Control of SRC Oncogenic Signalling by SLAP in CRC

### 3.1. SRC Tumour Activity in CRC

**Figure 2:**
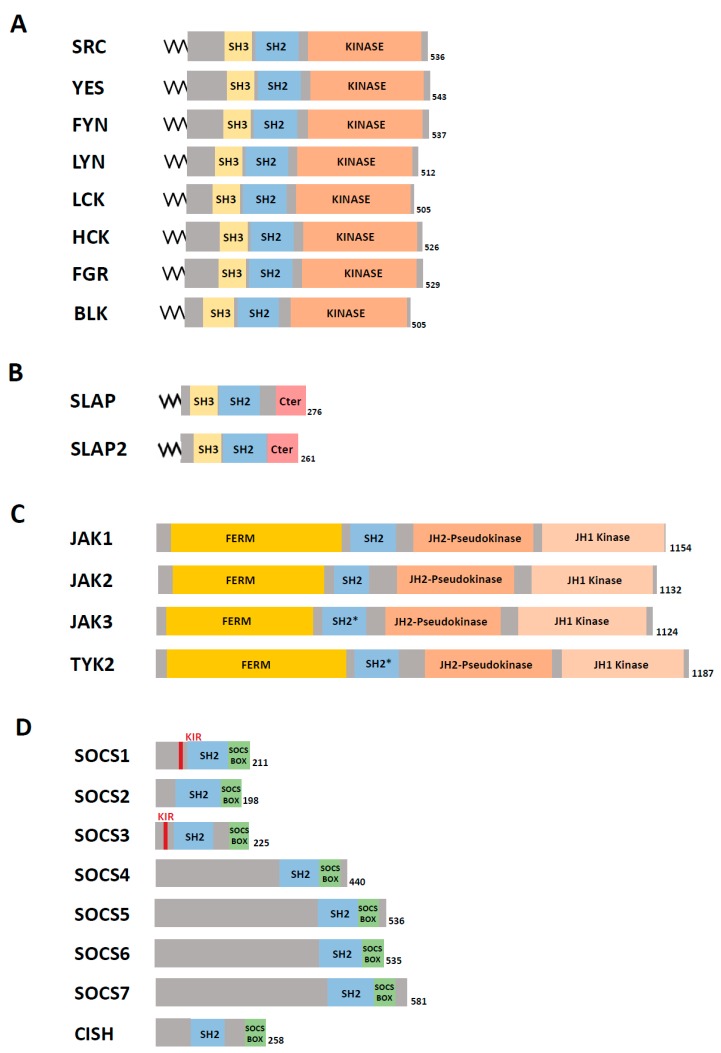
Modular structure of the SRC and Janus kinase (JAK) families of CTKs and of the Src-like adaptor protein (SLAP) and suppressor of cytokine signalling (SOCS) families of TK adaptors. Modular structure of the SRC (**A**), SLAP (**B**), JAK (**C**) and SOCS (**D**) families. The length (number of amino acids), specific homology domains (kinase inhibitory region (KIR); SRC Homology 2 (SH2); SH3; atypical SH2 domain SH2*; FERM, kinase domain JH1; pseudokinase JH2; SOCS BOX) and lipid acylation sites (/\/\) are indicated.

SRC, the first identified oncogene and TK, is a central transducer of cell signalling originating from a wide range of extracellular cues and is a master regulator of cell growth and adhesion [21]. SRC belongs to a family with eight members (SRC, YES, FYN, LYN, HCK, LCK, FGR and BLK) that is collectively named SRC Family Kinases (SFKs), the members of which share a similar modular structure (Figure 2A). In the intestinal epithelium, SRC drives intestinal stem/progenitor cell proliferation and tissue regeneration as well as tumourigenesis induced by aberrant WNT/β-catenin signalling in fruit fly and mouse models [22,23]. Hyperactive SRC displays prominent oncogenic activity in experimental CRC models [24]. SRC is frequently activated in CRC and higher SRC activity is common in metastases compared with the primary tumour [24,25]. Moreover, SRC activity is a marker of poor clinical prognosis in patients with CRC [26], favours therapeutic resistance [27] and is a potent driver of metastasis [24]. These cancer activities are linked to SRC’s capacity to promote CRC cells invasion and CSC activity. SRC plays additional important roles in CRC cell survival and in angiogenesis, which are necessary for tumour progression [24]. A SRC signature is primarily found in the CMS2 subtype, although SRC signalling may also be active in the CMS4 subtype on the basis of its mesenchymal and angiogenesis features [6]. Although less studied, FYN and YES display redundant functions with SRC in intestinal homeostasis and specific functions in tissue regeneration in mice [22]. YES also has a specific role in CRC cell invasion and CSC activity [28,29,30]. In agreement, its expression is a marker of poor prognosis in patients with CRC [24]. Although several SFK inhibitors have been developed, clinical trials in CRC have produced disappointing results probably because of the absence of selection of patients with SRC-dependent tumours and/or the drug’s inability to efficiently target SRC signalling [24,31,32,33]. Indeed, it is still unclear how SRC induces tumour progression in CRC [24]. Several studies indicate that SRC can affect major oncogenic pathways (e.g., the integrins, RTKs, WNT/β-catenin, Yes associated protein (YAP)/tafazzin (TAZ) and JAK/STAT signalling cascades) by direct or indirect mechanisms, and this may partly explain its tumour function [13,24,34,35]. However, phosphoproteomic analyses revealed that SRC phosphorylates several hundred substrates in CRC, including large groups of vesicular trafficking and mRNA binding proteins. This indicates that deregulation of protein trafficking and mRNA maturation may define additional important features of SRC signalling in CRC [34,36].

How SRC induces tumour formation remains mysterious, in part because SRC is infrequently mutated in human CRC (2.9% as reported in cbioportal.org) [24]. SRC contains an SH4 domain with a myristilation site for membrane localisation and activity, a unique intrinsically disordered region involved in protein dimerisation, an SH3 and an SH2 domain followed by the kinase domain flanked by two short regulatory regions (i.e., linker and C-terminus) (Figure 2A) [37,38,39,40]. Structural analyses show that upon phosphorylation of C-terminal Tyr530 by the C-terminal Src kinase (CSK), SRC is stabilised in a closed conformation through intramolecular interactions (SH2/C-terminus and SH3/linker) [37]. Disruption of these interactions derepresses SRC catalytic activity and may promote oncogenic properties, as exemplified by v-SRC that lost the C-terminal regulatory tail. In agreement, *Csk* ablation in the mouse intestine leads to development of hyperplasia throughout the intestinal epithelium, which involves SFK deregulation [41]. However, this mechanism does not operate in human cancer because SRC deregulation due to alteration of SRC C-terminal alteration or *CSK* inactivation has been rarely detected in human CRC. Actually, CSK was found upregulated in several CRC samples and anti-CSK autoantibodies were detected in these patients, which may define a novel biomarker of the disease [42]. The role of aberrant CSK expression in CRC is currently unknown. SRC is frequently upregulated in CRC, which primarily involves protein overexpression and/or gene amplification (10% of CRC) [43]. However, as SRC is physiologically tightly regulated, protein overexpression is not sufficient to promote its oncogenic activity. It was reported that a complex epigenetic mechanism modulates the CRC cells’ capacity to regulate SRC catalytic activity via CSK membrane delocalisation. Consequently, upregulated SRC displays high TK activity in metastatic cells, promoting invasive capacities of CRC cells [44,45,46]. However, this mechanism alone may not be sufficient to explain SRC tumour activity observed in experimental animal models and patients.

### 3.2. SLAP Tumour Suppressor Activity in CRC

**Figure 3:**
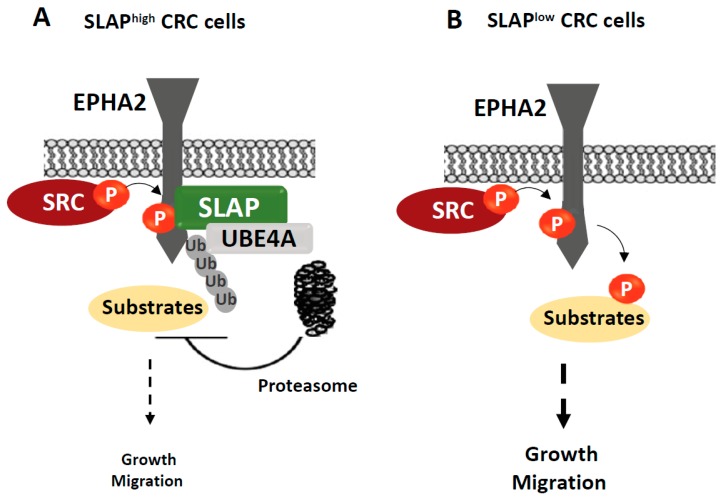
Model depicting how SLAP counteracts SRC signalling in CRC cells. (**A**) In CRC cells that express SLAP, SRC phosphorylates EPHA2 on Tyr594. This promotes EPHA2/SLAP/UBE4A complex formation and consequently EPHA2 proteasomal degradation and inhibition of SRC invasive signalling. (**B**) SLAP downregulation in CRC cells leads to aberrant EPHA2 expression and SRC-dependent EPHA2 signalling, which promote tumour cell growth and migration. Ub, ubiquitin; P, phosphorylation.

In vertebrates, the *SLA* gene, which encodes SLAP, has emerged from *SRC* duplication [9] and SLAP is composed of an N-terminal region similar to that of SRC (i.e., a short myristoylated sequence followed by the SH2 and SH3 domains) and a unique C-terminus with binding affinity to the ubiquitination factor Casitas B-lineage lymphoma proto-oncogene CBL (Figure 2B). SLAP is strongly expressed in haematopoietic cells, epithelial intestine, lung and brain [47,48]. SLAP2, the other member of the SLAP family, is preferentially expressed in the haematopoietic tissue and the lungs [47]. *Slap* inactivation in mice revealed its important role in the development and activity of lymphocytes, where it is highly expressed. Mechanistically, SLAP docks CBL to tyrosine phosphorylated substrates for degradation and thus dampens the receptor signalling needed for lymphocyte development and activity [49,50]. Conversely, the SLAP role in nonimmune cells is still not clear. We have previously shown that SLAP controls cell proliferation and morphology in murine embryonic fibroblasts, most likely by competing with SRC signalling components for TK binding [51]. SLAP can efficiently counteract SRC oncogenic activity in these cells [52,53]. Moreover, SLAP displays a prominent tumour suppressive function in human colonic epithelial cells by controlling essential SRC tumour-promoting activities described in CRC, including tumour cell growth and migration [54]. In agreement, SLAP is also abundantly expressed in murine intestine and human colon epithelium, where its expression level is associated with epithelial cell differentiation. Notably, *SLA* mRNA expression is frequently downregulated in CRC tissues compared with healthy peritumoural tissues (Table 1). The underlying mechanism of this inhibition is unknown. Functionally, *SLA* silencing in early stage CRC cells promotes tumour formation and colon liver metastasis in nude mice, while SLAP overexpression reduces tumour growth. In addition, SLAP silencing increases intestinal tumour initiation and progression in *Apc^Δ14/+^* transgenic mice that carry a heterozygous mutation of the APC tumour-suppressor gene and consequently develop WNT-pathway-driven intestinal tumours. Compelling evidence indicates that in human CRC cells, SLAP acts as a tumour suppressor by controlling SRC oncogenic activity. For instance, SLAP overexpression reduces SRC cancer activities, while its inactivation potentiates this malignant process. How SLAP counteracts SRC signalling in CRC tumours remains to be clarified, but several mechanisms can be envisaged. While SLAP does not inhibit SRC nor the overall protein tyrosine phosphorylation induced by SRC expression, it can promote the destabilisation of critical SRC substrates upon their aberrant phosphorylation to limit the oncogenic signalling cascade. In agreement, we reported that SLAP attenuates tumour cell dissemination via destabilisation of the adhesive receptor EPHA2 (Figure 3). This implicates the association with the ubiquitination factor UBE4A, which was previously shown to be involved in Crohn’s disease [55]. Nevertheless, SLAP interatomic analysis in CRC cells suggests that SLAP may act through additional mechanisms to be characterised [54].

**Figure 4:**
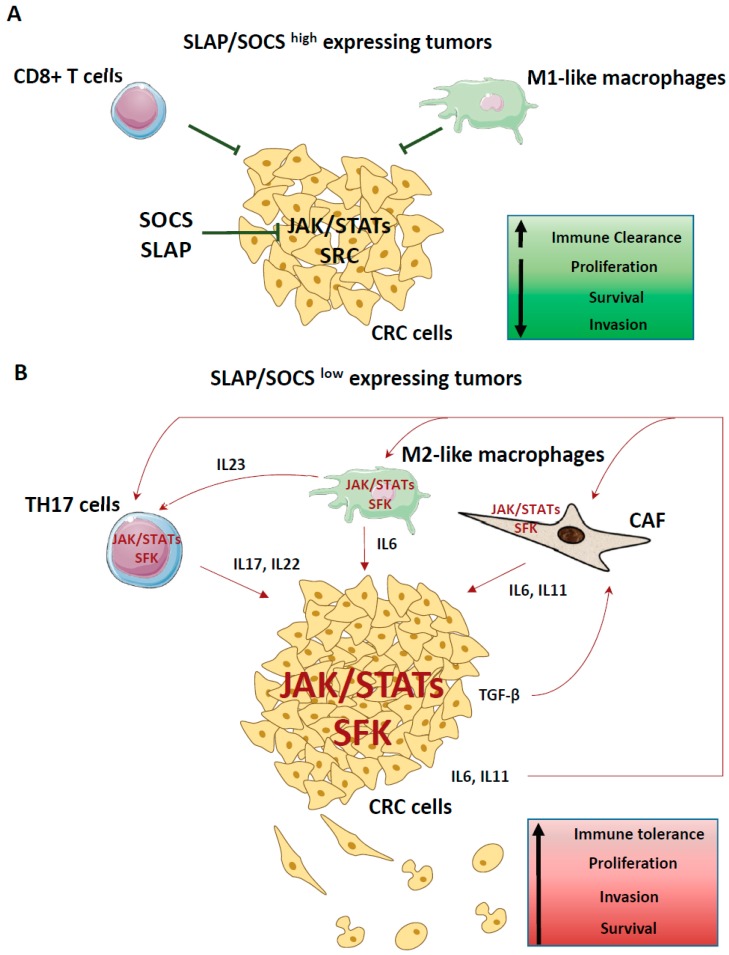
Model describing how SLAP and SOCS adaptors modulate SRC- and JAK-dependent tumour activities in CRC. (**A**) High SLAP and SOCS expression level in CRC limits JAK inflammatory and SRC/JAK tumour signalling, resulting in increased immune clearance and reduced tumour cell growth and invasion. (**B**) Downregulation of these adaptors results in enhanced JAK inflammatory and JAK/SRC tumour signalling and, consequently, higher tumour immune tolerance and tumour cell growth and invasion.

While not experimentally explored, SLAP could act on the stromal component in CRC (Figure 4). Indeed, the SFK haemopoietic cell kinase (HCK) was shown to increase myeloid-cell-mediated colon cancer progression in experimental CRC models. This effect was associated with alternative macrophage polarisation and the accumulation of cytokines of the IL-6/lL-11 family that drive a STAT3-dependent growth response in CRC cells [75]. Consistently, HCK genetic ablation or pharmacological inhibition reduced tumour development in mouse models. The relevance of HCK protumoural function in human pathology has been supported by the correlation between increased HCK level in tumour leukocytes and reduced survival of patients with CRC [75,76]. Interestingly, in macrophages, SLAP and SLAP2 promote CBL-dependent proteasomal degradation of the RTK colony stimulating factor 1 receptor (CSF1R), the upstream activator of HCK in these cells [77,78]. Consequently, SLAP inhibition in this compartment could aggravate HCK protumour activity. Likewise, SLAP negatively regulates platelet derived growth factor receptor (PDGFR) signalling [51], which plays a role in stromal cell activities, such as cancer-associated fibroblasts or endothelial cells. Therefore, SLAP could have a function in tumour angiogenesis and metastasis development. Clearly, the stromal contribution in SLAP tumour-suppressor function deserves further investigations.

## 4. The Control of JAK Oncogenic Signalling by SOCS in CRC

### 4.1. JAK/STAT Activities in CRC

**Figure 5:**
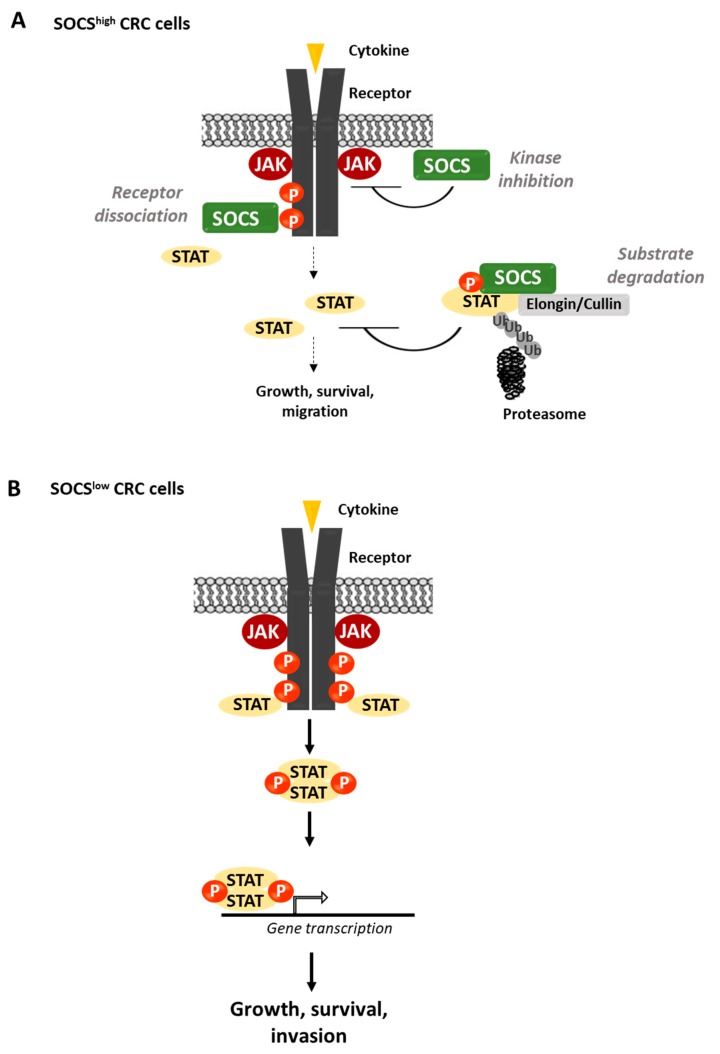
Model depicting how SOCSs can counteract JAK/signal transducer and activator of transcription (STAT) signalling in CRC cells. (**A**) In CRC cells that express SOCS, these adaptors can inhibit STAT phosphorylation by preventing its association with the receptor, promoting its proteasomal degradation or inhibiting JAK kinase activity through direct binding. (**B**) SOCS downregulation in CRC cells, by gene promoter methylation or epigenetic mechanisms, leads to aberrant JAK/STAT signalling that promotes tumour cell growth and migration. Ub, ubiquitin; P, phosphorylation.

The JAK CTKs are major transducers of cytokine and interferon signals to regulate tissue inflammation and immune response [79,80,81]. Mechanistically, JAK proteins constitutively associate with cytokine and growth factor receptors (Figure 5B) [81]. Upon ligand binding, receptor dimerisation confers JAK activation by disrupting the intramolecular interaction between the JH1 kinase domain and its pseudokinase domain JH2 (Figure 2C). JAK can then phosphorylate the STAT transcription factors (Figure 5B). Tyrosine phosphorylation enables STAT dimerisation via SH2 and its nuclear translocation to bind to DNA consensus motifs and elicit specific transcriptional responses [81]. The JAK/STAT family includes four CTKs, (JAK1–3 and TYK2 [82,83,84]) (Figure 2C) and eight STAT proteins (STAT1–4, 6 and 7 and STAT5A and B). STAT1 and 2 are mainly involved in immune responses. The combination of JAK and STAT proteins confers signalling specificity induced by upstream cytokines. Interestingly, JAK somatic mutations, including some frequent mutations in the JH2 regulatory domain, have been identified in myeloproliferative diseases where these mutants display a protumour function [85,86,87,88,89,90,91,92]. In CRC, JAK and STAT components are not frequently mutated [93,94], while excessive JAK/STAT activity is found in CRC and more specifically in the CMS1 subtype [6]. Recent studies in a small cohort of CRC specimens identified a JAK1 inactivating mutation in MSI+ CRC (20%) that was associated with good prognosis [95,96]. Conversely, JAK2 genetic variation was associated with higher CRC risk in two distinct cohorts [63,97]. It is not clear whether alteration of other JAK/STAT components has a prognostic role in patients with CRC. This may depend on the tumour subtype, as suggested for JAK1. For instance, STAT3 activity is associated with elevated malignancy and invasive behaviour of CRC cells [98,99,100,101], while it has anticancer activity in the *Apc^Min/+^* mouse model of intestinal tumourigenesis [102,103]. On the other hand, the prognostic value of STAT3 activity in patients with CRC is conflicting. Additional studies are needed to clarify this discrepancy. The physiological role of JAK/STAT signalling in the intestine is largely unknown and genetic evidence for its role in mouse intestinal regeneration is lacking, mainly because of the strong defects observed upon constitutive gene ablation [104,105,106,107,108,109,110,111]. Conditional ablation of *Tyk2* in the mouse intestine revealed that this JAK member has an essential role in protection from acute colitis transduced by the IL-22 signal [112]. Genetic experiments in flies demonstrated an important noncell autonomous role of JAK/STAT activity in the proliferation of intestinal epithelial stem cells after tissue damage or infection. This occurs via secretion of the IL-6 ortholog Upd from neighbouring epithelial cells [113,114,115,116,117]. Surprisingly, in transgenic mouse models, overexpression of GP130, the coreceptor for IL-6 cytokine, in the intestinal epithelium triggers SFK-dependent activation of YAP and Notch signalling to control tissue growth and regeneration, in the absence of its canonical STAT3 effector [118]. Therefore, several mechanisms may be involved in intestinal regeneration depending on the nature of the tissue injury.

JAK/STAT activities in CRC primarily originate from extrinsic inflammatory signals (Figure 4B). Indeed, inflammation can increase the risk of CRC, and JAK/STAT signalling plays an important role in this process. This is particularly true for chronic inflammatory bowel diseases where IL-6 is a critical tumour promoter during early colitis-associated tumourigenesis [119]. Specifically, IL-6 produced by lamina propria myeloid cells induces STAT3-dependent proliferation and survival of intestinal CSCs. A similar JAK/STAT inflammatory function is involved in sporadic CRC, upon localised loss of the intestinal epithelial barrier. Mechanistically, invasion of the colon microbiota activates IL-23-synthesising myeloid cells and expands tumour-resident IL-17-producing T lymphocytes to induce aberrant epithelial cell proliferation and adenoma formation [120]. In agreement, high levels of IL-17 and IL-23 are bad prognostic markers in patients with early CRC [121,122,123]. Similarly, high stromal expression of TGF-β during CRC progression stimulates IL-11 production by cancer-associated fibroblasts and subsequently GP130/JAK/STAT signalling in CRC cells, leading to augmented cell survival and metastatic behaviour [124]. Interestingly, JAK/STAT3 activity is also an intrinsic property of CRC cells. For instance, aberrant SRC activity may induce excessive JAK/STAT signalling by direct phosphorylation. In support of this notion, STAT3 is heavily phosphorylated in SRC-transformed fibroblasts and its activity is needed for SRC transforming activity [125]. Moreover, loss of the adenomatous polyposis coli (APC) tumour suppressor in mice results in GP130 upregulation, inducing SRC/YAP and JAK/STAT3 pathways that are needed for tumour formation [35]. In agreement, inhibition of GP130/JAK/STAT3 signalling by genetic or pharmacological means prevents WNT/β-catenin-mediated intestinal tumour growth and intestine regeneration in mice [126]. The relevance of these finding in sporadic CRC was provided by the reduced tumour xenograft development in nude mice upon pharmacological inhibition of the GP130/JAK/STAT3 pathway. In nude mice, which are immunodepressed, tumour cell grafting did not induce an overt inflammatory response; therefore, the observed effect was due to inhibition of the GP130/JAK/STAT3 pathway in cancer cells and not in inflammatory cells [127]. These important results suggest the therapeutic utility of targeting this JAK/STAT activity in CRC. Small JAK inhibitors are currently being tested in clinical trials in patients with advanced CRC [96].

### 4.2. SOCSs Activity in CRC

SOCS adaptor proteins are important inhibitors of JAK/STAT signalling. They emerged early during evolution together with the appearance of JAK/STAT signalling components [128,129,130]. In mammals, there are eight SOCS members (SOCS1–7 and CIS) with a similar modular structure: a central SH2 domain that shows homology with the STAT SH2, and a SOCS box that interacts with Elongin and Cullin to confer E3 ligase activity (Figure 2D). SOCS1 and SOCS3 also possess an N-terminal kinase-inhibitory region (KIR) involved in JAK catalytic inhibition. SOCSs are induced by cytokine stimulation, mostly via STAT proteins, to control the duration of kinase signalling. Mechanistically, they can induce degradation of cytokine receptors, JAK proteins and specific components of the targeted signalling cascade, such as IRS1/2 of the insulin pathway and MAL of the toll-like receptor pathway. SOCSs can also inhibit STAT recruitment to cytokine receptors through a competitive mechanism and JAK activity by direct binding (Figure 5A). Finally, their function is not restricted to inflammatory signals because SOCS proteins also interact with growth factor receptors (e.g., EGFR, IGF1R and growth hormone (GH) receptor) to control their activity [18]. Their physiological role in the intestine has not been explored yet, probably due to the major defects in immunity and tissue growth observed upon gene ablation in mice [19]. However, it was reported that *Socs2*^−/−^ mice develop gigantism due to enhanced responses to GH, including in the intestinal epithelium. This establishes SOCS2 as a GH-inducible inhibitor of intestinal growth [131].

In CRC, SOCS expression alteration by genetic and epigenetic mechanisms can have an important impact on tumour formation (Table 1). Notably, the *SOCS1* gene is frequently silenced by hypermethylation of its promoter, specifically in the CMS1 subtype, where SOCS1 is a marker of the CpG island methylator phenotype (CIMP) class with a better survival rate [132]. Consistent with a possible SOCS1 tumour-promoting role, colon carcinogenesis induced by 1,2-dimethylhydrazine plus dextran sulphate sodium was reduced in *Socs1*^−^^/^^−^ mice. This effect was attributed to enhanced macrophage activity and tumour-specific cytotoxic T-cell activity upon *Socs1* inactivation [133]. SOCS1 expression in some CRC cell lines can increase their transforming properties [134]. On the other hand, other studies revealed SOCS1 tumour-suppressive functions in CRC [57,135]. For instance, SOCS1 prevents chronic-inflammation-mediated carcinogenesis in mice. Specifically, *Socs1*^−/−^ mice spontaneously develop intestinal tumours in an IFNγ/STAT1-dependent manner, suggesting that SOCS1 controls the chronic inflammatory JAK/STAT1 signalling needed for CRC development [135]. Moreover, a functional analysis suggested that SOCS1 expression controls CRC metastatic progression, possibly through destabilisation of metastatic inducers. In addition, CSC activity in CRC cells can be maintained through miR-196b-5p-dependent SOCS1 and 3 silencing to sustain high STAT3 activity [64]. Overall, these results suggest that SOCS1 may have both protumour and antitumour activities, depending on the tumour genetic profile and the microenvironment.

Unlike SOCS1, other SOCS proteins display unambiguous tumour-suppressor function in CRC (Table 1 and Figure 5B). For instance, downregulation of SOCS 2, 3 and 6 is associated with bad prognosis [65,68]. *Socs2* inactivation promotes intestinal tumour formation in *Apc^Min/+^* and GH transgenic mice [136]. *Socs3* inactivation also is associated with STAT5-dependent CRC metastatic progression [137]. SOCS3 can limit inflammation-associated tumourigenesis in the colon by inactivating STAT3 and NF-κB [138]. Moreover, downregulation of SOCS5–7 mediated by miR-885-5p [70] or miR-301a [74] upregulation increases CRC cell proliferation and migration. Conversely, SOCS3 overexpression reduces CRC cells’ proliferative and invasive properties [68]. Interestingly, some evidence highlights additional mechanisms for SOCS activities in CRC. For instance, SOCS3 overexpression can inhibit the TGF-β signalling in CRC cells [139], and a proteomic analysis identified EPHA2 as an important SOCS2 target [140]. Due to the prominent tumour role of EPHA2 in CRC [54], this mechanism could be involved in SOCS2 antitumour activity in CRC.

## 5. Discussion

All these data reveal an important but still rather unexplored mechanism for the control of TK oncogenic activities in CRC. Therefore, SLAP and SOCS expression status in CRC, both in the tumour compartment and the microenvironment, may have a significant impact on the tumour response to anti-TK therapies. For instance, a low SLAP expression level may enhance SRC oncogenic activity in CRC and could become more responsive to SRC-like inhibitors. Conversely, high SLAP expression could limit SRC oncogenic signalling and, therefore, tumours could be less responsive to SRC-like inhibitors, despite the high aberrant SRC activity. Consistent with this idea, we showed that CRC cells are more responsive to SRC-like inhibitors upon *SLA* silencing [54]. Therefore, the level of SRC expression alone may not be a good predictor of CRC response to such inhibitors. This may explain, at least in part, the disappointing results of these drugs in CRC. This notion may also apply to any additional SLAP oncogenic targets of therapeutic interest in this cancer. Finally, any mechanism that restores SLAP expression in CRC might represent an interesting therapeutic strategy.

Similarly, understanding how SOCS regulates JAK/STAT signalling may lead to effective therapeutic strategies in CRC. For instance, low SOCS expression levels exacerbate JAK/STAT signalling in CRC cells (Figure 4B), and consequently, these tumours may be responsive to GP130/JAK/STAT signalling inhibition. As JAK inhibitors are currently in clinical trials in CRC [96], it would be interesting to correlate the tumour SOCS protein levels and the tumour response to these drugs. These adaptors are frequently inactivated via a methylation-dependent mechanism. Restoring their expression level using demethylation drugs may represent a potential antitumour therapeutic strategy. Moreover, SOCS1 and 3 directly inhibit JAK catalytic activities. This specific mechanism may pave the way for the development of more specific JAK inhibitors. However, due to the complex role of SOCS1 in CRC progression, this protein may not be a good predictor of tumour response to JAK inhibitors in this cancer. Similarly, JAK/STAT signalling is a specific feature of the CMS1 subtype that displays strong immune reactivity [6]. In agreement, immune-checkpoint-based therapies gave promising results in this CRC group. However, it has been proposed that JAK1/2 loss-of-function mutations are implicated in resistance to anti-programmed death protein 1 (PD-1) therapy including in MSI+ CRC [141]. Therefore, it should be important to test whether the level of specific SOCS proteins can also be involved in resistance to anti-PD-1 therapy in CRC and whether SOCS inhibitors could alleviate this therapeutic resistance.

## 6. Conclusions

Overall, SLAP and SOCS adaptors have emerged as important mechanisms in the regulation of TK oncogenic activities in CRC. However, there is still much to learn about how they precisely control these signalling cascades in CRC. For instance, genetically modified mouse models targeting these signalling components in the intestine could be a good biological tool to address this complex question. Moreover, a thorough analysis of their protein level in the tumour and its microenvironment in large cohorts of patients with CRC could clarify their tumour role in CRC. Finally, molecular studies may reveal additional mechanisms by which these adaptors control oncogenic signalling in this cancer. These future studies may allow improving TK-based therapies and better identifying patients with CRC who might respond to SRC or JAK inhibitors.

## Figures and Tables

**Table 1:** Deregulation of SLAP and SOCS proteins in colorectal cancer (CRC) and underlying mechanisms.

Adaptor	Status in CRC	References
SLAP	Downregulation	[56]
		[54]
SOCS1	Downregulation	[57]
	Hypermethylation	[58,59,60,61,62]
	SNPs association with CRC	[63]
	Downregulation by miRNA	[64]
SOCS2	SNPs association with CRC	[63]
	Downregulation	[65]
	Upregulation	[66]
SOCS3	Hypermethylation	[67]
	Downregulation	[68,69]
	Downregulation by miRNA	[64]
SOCS5	Downregulation by miRNA	[70,71]
SOCS6	Gene loss	[72]
	Hypermethylation	[73]
	Downregulation	[65]
	Downregulation by miRNA	[70,74]
SOCS7	Hypermethylation	[67]
	Downregulation by miRNA	[70]
